# How deep is deep enough? Analysis of sea turtle eggs nest relocation procedure at Chagar Hutang Turtle Sanctuary

**DOI:** 10.1016/j.sjbs.2021.05.021

**Published:** 2021-05-13

**Authors:** Siti Najwa-Sawawi, Nur Munira Azman, Mohd Uzair Rusli, Amirrudin Ahmad, Muhammad Fahmi-Ahmad, Nik Fadzly

**Affiliations:** a School of Biological Sciences, Universiti Sains Malaysia, 11800 Minden, Penang, Malaysia; b School of Marine and Environmental Sciences, Universiti Malaysia Terengganu, 21030 Kuala Nerus, Terengganu, Malaysia; c Institute of Oceanography and Environment, Universiti Malaysia Terengganu, 21030 Kuala Nerus, Terengganu, Malaysia

**Keywords:** Hatchery management, Hatching success, Conservation, Endangered species, South China Sea

## Abstract

Sea turtle eggs incubation involves natural and artificial incubation of eggs, and indeed the depth will be varied and presumably affect the development of hatchlings. For nest relocation, the researcher needs to decide on the depth to incubate the eggs. Sea turtle eggs clutches may vary between 40 and 120 eggs for the green turtle, thus using a single value as the standard procedure might affect the quality of hatchlings. Here we quantify the dimension of the natural (in-situ) nest constructed by the nester and the artificial (ex-situ) built by our ranger during nest relocation. We suggest a linear regression calculation of Y = 0.2366X + 59.3267, better predict a more accurate nest depth based on the number of eggs to imitate the natural nest.

## 1 Introduction

Nest relocation, or typically refers as *ex-situ* incubation, is one of the conservation techniques that has been regularly implemented to reduce threats towards the nest (Turkozan et al., 2011). Although the sea turtle eggs should be left undisturbed to be incubated in their natural nest (Mortimer, 1999), the relocation of egg clutches should be done due to the swift coastal development and rapid construction of human settlement near the beach(Hewavisenthi, 2001). However, there are many potential dangers in implementing the *ex-situ* conservation method if the nest are not imitating the natural ones as closely as possible (Pritchard, 1980). The *ex-situ* conservation could do more harm than good if the management does not consider the relocation area, nest depth, nest shape, temperature, and precipitation during the translocation process (Hewavisenthi, 2001). More recently, Tanabe et al. (2021) suggested that relocation should only be implemented on clutches with a high potential to be disrupted or with a low chance of survival if left in situ.

The question of the effectiveness of *ex-situ* conservation is improving since more information is gained from the latest hatchery management studies (Nastiti and Wiadnyana, 2013). Profound comprehension regarding the beach composition (Mortimer, 1990) and nest shape, including the depth (Koch et al., 2007), is crucial for the survival of eggs during the incubation period.

Environmental parameters play a crucial role in determining the hatchling quantity and quality of sea turtle, especially temperature and precipitation. According to Fleming et al. (2020), the increment of incubation temperature adversely impacts the hatchling rate by increasing the predation risk. Besides, grain size also could enhance the development of eggs during incubation (Yalcın-Ozdilek et al., 2007) and vegetation control which influences the sea turtle nest productivity on the beach (Conrad et al., 2011). For ex-situ conservation, the nest must have optimum nest depth and shape to maintain the nest productivity. Thus, it is crucial to keep the suitability of surrounding parameters for both in situ and ex situ conservation to enhance the hatchling success of sea turtle.

As the sea turtles extend no maternal care towards their eggs or hatchlings after oviposition took place (Koch et al., 2007), the progress of embryo development entirely depends on the nest environments; where there is an exchange of oxygen, moisture and heat among incubated eggs (Ackerman, 1997, Miller et al., 2017). Incubation temperature and humidity that influence the nest depth can strongly affect the hatching success and sex ratio of sea turtle (Martins et al., 2007). A researcher or conservationist must know the nest shape and dimension of the sea turtle, especially for the ex situ conservation method. The relocated eggs must be implanted inside a natural-resembling nest in the hatchery. In this short communication, we aim to 1) measure the nest dimension of green turtle natural nest and 2) to describe and compare the relationship between nest depth and hatching success of green sea turtles from natural and artificial nest.

## 2 Materials and method

### 2.1 Study area

**Fig. 1:**
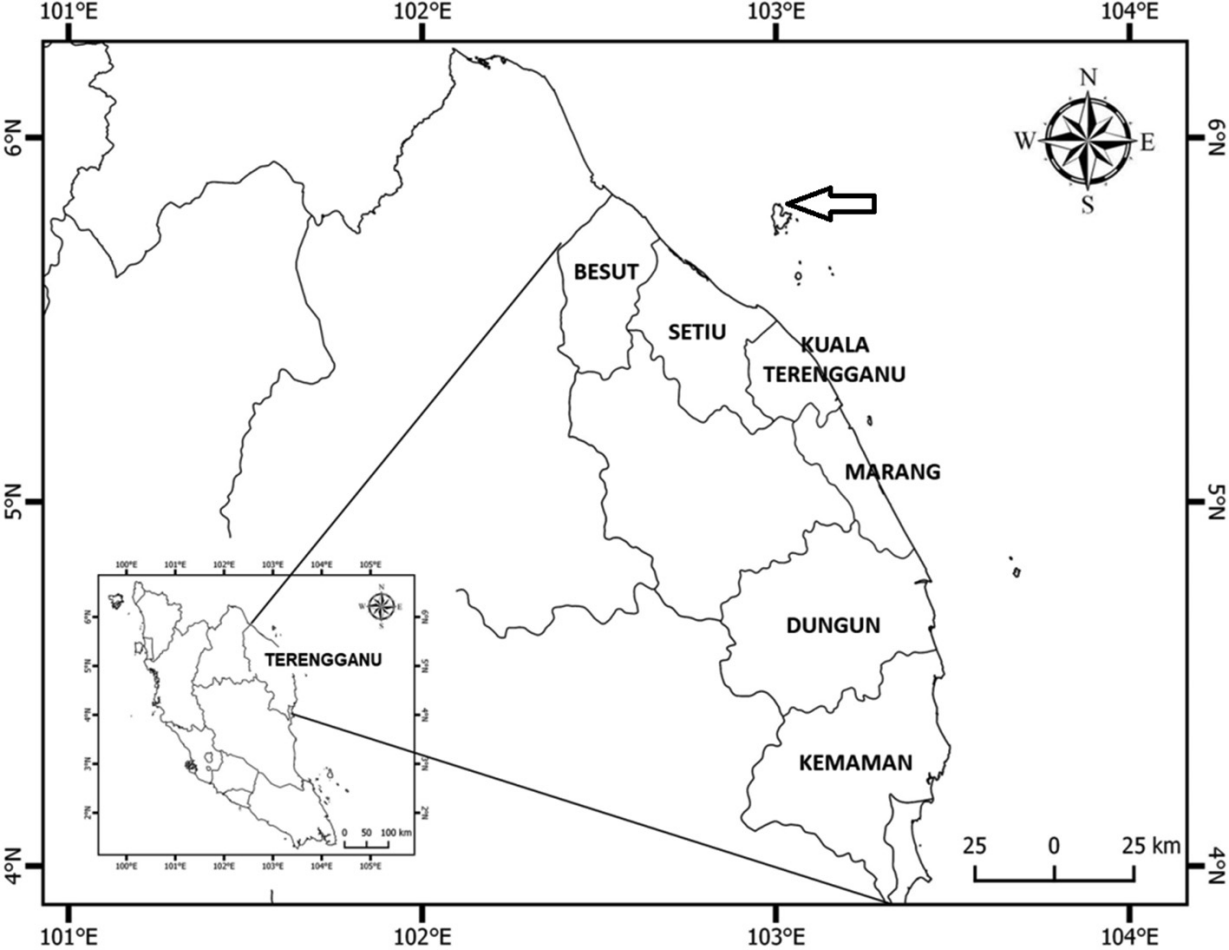
Map of Chagar Hutang Turtle Sanctuary in Pulau Redang, Terengganu.

**Fig. 2:**
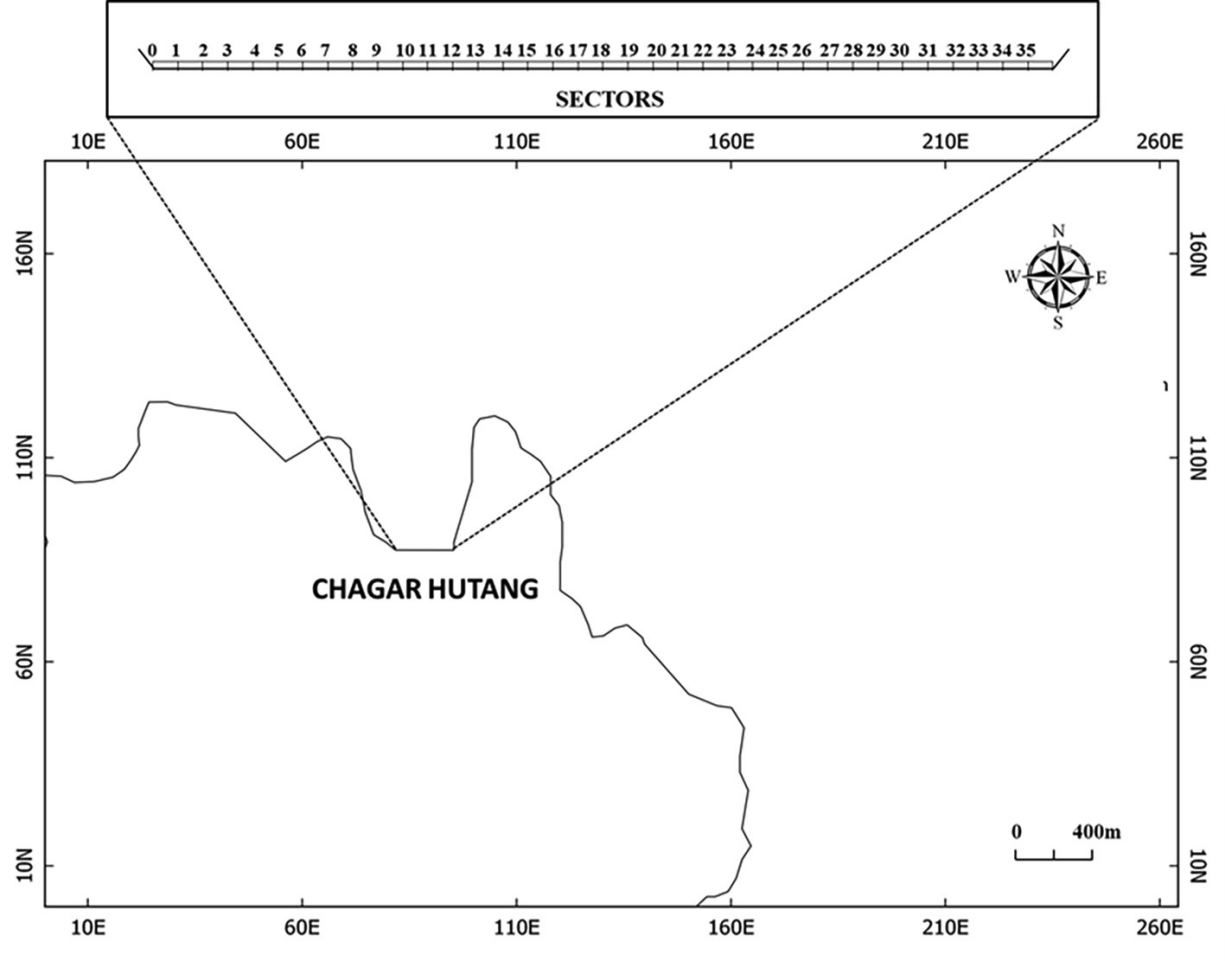
Close up of Chagar Hutang with 35 sectors. The inset map shows the satellite image from the Google Maps of Chagar Hutang.

Chagar Hutang Turtle Sanctuary (CHTS) is located in the northern part of Pulau Redang in Terengganu state, east coast of Peninsular Malaysia (Fig. 1). CHTS is known to host the most favoured beach for the green turtle nesting in Peninsular Malaysia, with approximately hosts 700–1500 nests per season. Besides CHTS in Pulau Redang archipelago, there are two other central locations for turtle nesting in Pulau Redang: Mak Kepit and Mak Simpan, which are directly managed by the Department of Fisheries (DoF). CHTS is operated by the Sea Turtle Research Unit (SEATRU) of University Malaysia Terengganu (UMT) and strictly prohibited for the public with several occasion visit authorised by management. The 350 m long beach at CHTS has been divided into 35 sectors equally (Fig. 2) and implemented both *in-situ* (circa 70%) and *ex-situ* incubation (under several occasions of unsuitable nest location by nester). Chagar Hutang beach has fine sand, surrounded by vegetation and a gradual slope. The beach morphology believed to be the attraction factor for the green turtle to nest.

### 2.2 Beach patrolling and *in-situ* nest management

**Fig. 3:**
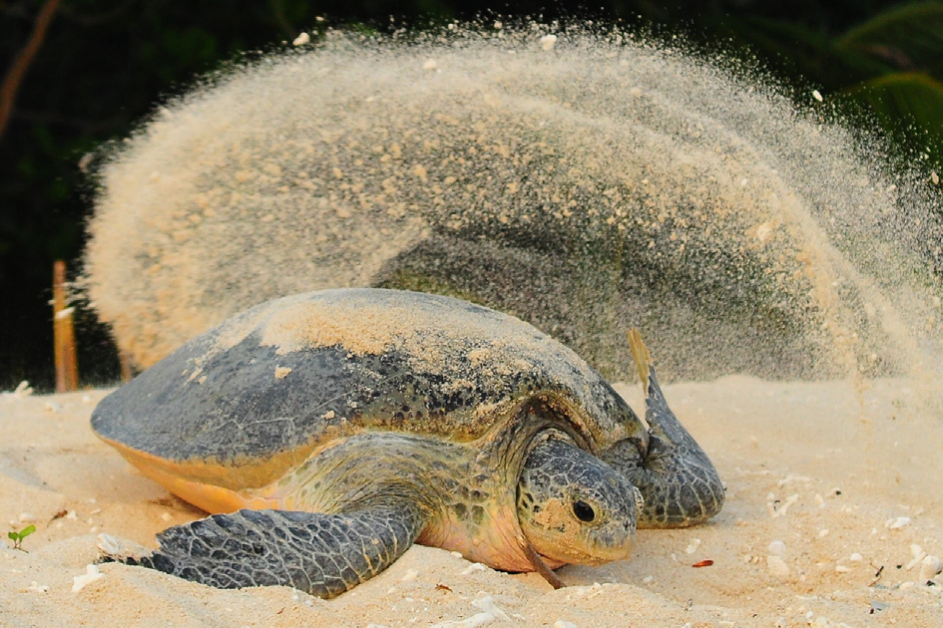
A Green Turtle covering the nest site after oviposition.

Night beach patrolling was conducted every night from 1800 to 0600 h while the excavation process was conducted during day time to monitor incubating eggs, hatchlings production and nest condition. The nesting activities are observed approximately 5–10 m by rangers and volunteers to avoid any interruption during the nesting attempt (Fig. 3). When oviposition occurs, a marking string attached to a stick was placed near the nest without distracting the remaining processes. Upon egg-laying completion, nesting females will be tagged and measured their body for long term monitoring of the population.

### 2.3 *Ex-situ* eggs relocation

**Fig. 4:**
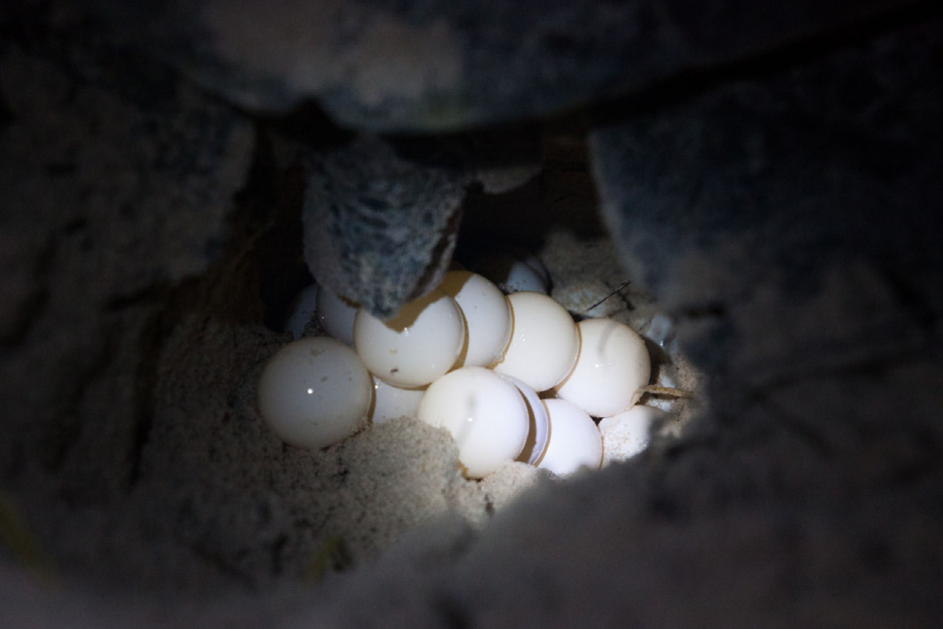
A close up of the oviposition process.

**Fig. 5:**
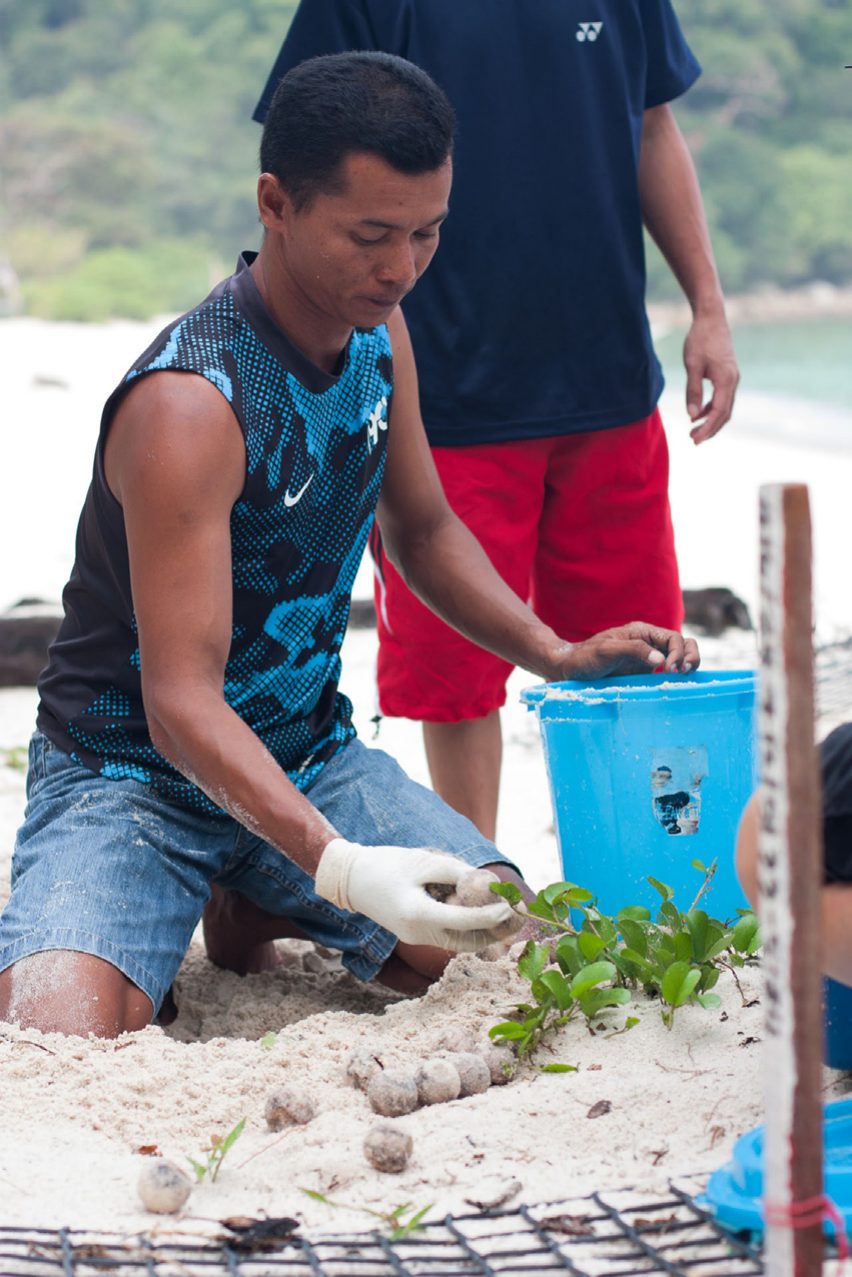
A hatchery worker carefully excavating and transporting the eggs.

If the oviposition occurred at the vulnerable sectors, the nest should be relocated. Sea turtle eggs will be placed inside a basin upon oviposition by nesting females to be relocated to another place that identified as safe and has a higher incubation success (Fig. 4 and Fig. 5). The experienced SEATRU rangers constructed the artificial nest. The nest depth typically constructed varied between 80 cm and 100 cm (measures with their entire hand length) depending on the number of eggs. The nesting data, date, time, nest number, number of eggs and tagging number was taken, and the nest then was left for incubation for 45 to 60 days before the excavation process was conducted.

### 2.4 Post-hatching excavation process

On the 45th days of incubation, an initial nest inspection was conducted to observe the nest condition. If the eggs have not hatched, or the hatchlings were still at the bottom of the nest, they will be covered back. The exact process will be repeated after three days gap until all the hatchling successfully emerged. Afterwards, another excavation will be conducted to quantify the number of hatched and unhatched eggs and the number of dead hatchlings. To determine the number of hatched eggs, the fragmented shell will be counted (>50% intact based on Miller, 1999). Next, the hatching success, emergence success and mortality rate will be determined.

**Fig. 6:**
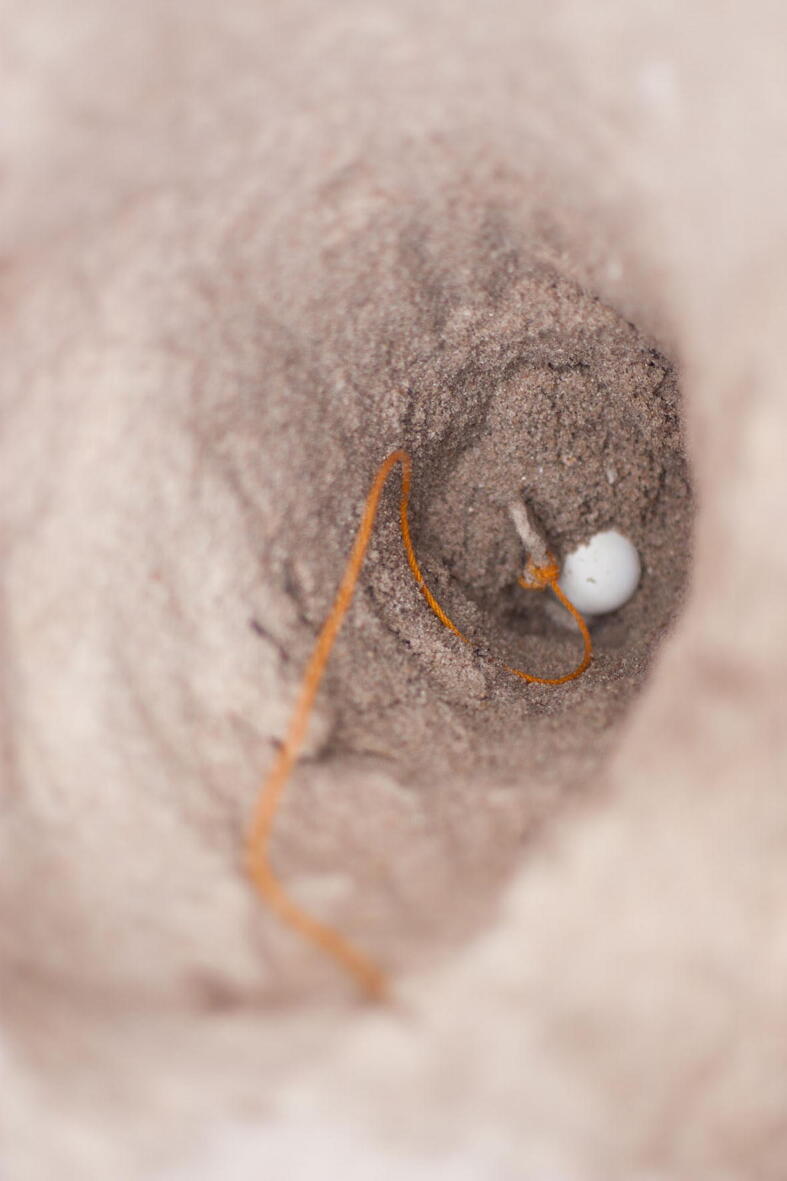
Measurement of the depth using a weighted line.

**Fig. 7:**
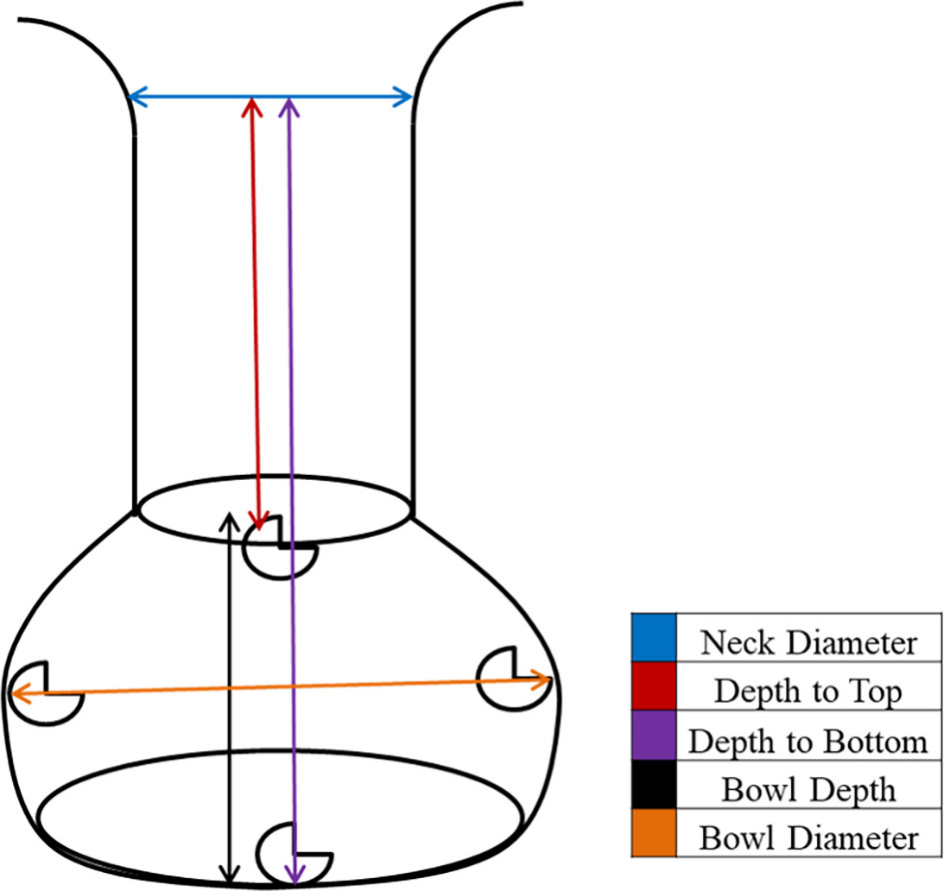
The illustration of the nest measurement variables.

During the nest dimension measurement, the nest was carefully dug during the excavation process. The top of the bowl nest shape was measured when the first top eggs shell was. Then the nest was carefully dug, and clutch residue will be removed and marked on the left and right wall of the nest chamber. Only two persons were assigned to dig and measure the nest during excavation to avoid parallax error. We measured; the diameter of the flask neck, the depth to bottom, the depth to top, the depth of the bowl, and the bowl's diameter (Figs. 6 and 7).

### 2.5 Data analysis

The hatching success was calculated using the following formulas and definitions, as Miller (1999) suggested.

$$\text{Hatching Success}\;\left(\text{HS}\right)\;\;\text{=}\;\;\text{S/C}\;\times \;\text{100}$$

where S is the number of hatched eggs collected, and C is the total number of eggs. All parameters were tested for normality before proceeding with parametric tests. Descriptive statistics (mean and standard deviation) was used to describe the nest characteristics. All statistical analyses were made using PAST (Paleontological Statistics) software version 3.0 and Microsoft Excel 2010. Regression analysis was used to determine the relationship between the number of eggs with depth to the bottom of the nest and to calculate the suitable depth for a certain amount of eggs.

## 3 Result

### 3.1 *In-situ* nest dimension measurement

In 2017, 968 nests were recorded at CHTS, and 36% of nests were relocated (*ex-situ*) while the rest were left incubated naturally (*in-situ*). In this study, 32 *in-situ* nests were selected and excavated to measure the dimension of the nest chamber. From this study, the results show that the average *in-situ* nest depth of the green turtle was 79.4 ± 12.4 cm and ranging from 61.1 cm to 101.2 cm. The highest eggs per clutch were recorded at 110 eggs with a nest depth of 92.1 cm, while the lowest was with 20 eggs with a nest depth of 76.4 cm.

**Fig. 8:**
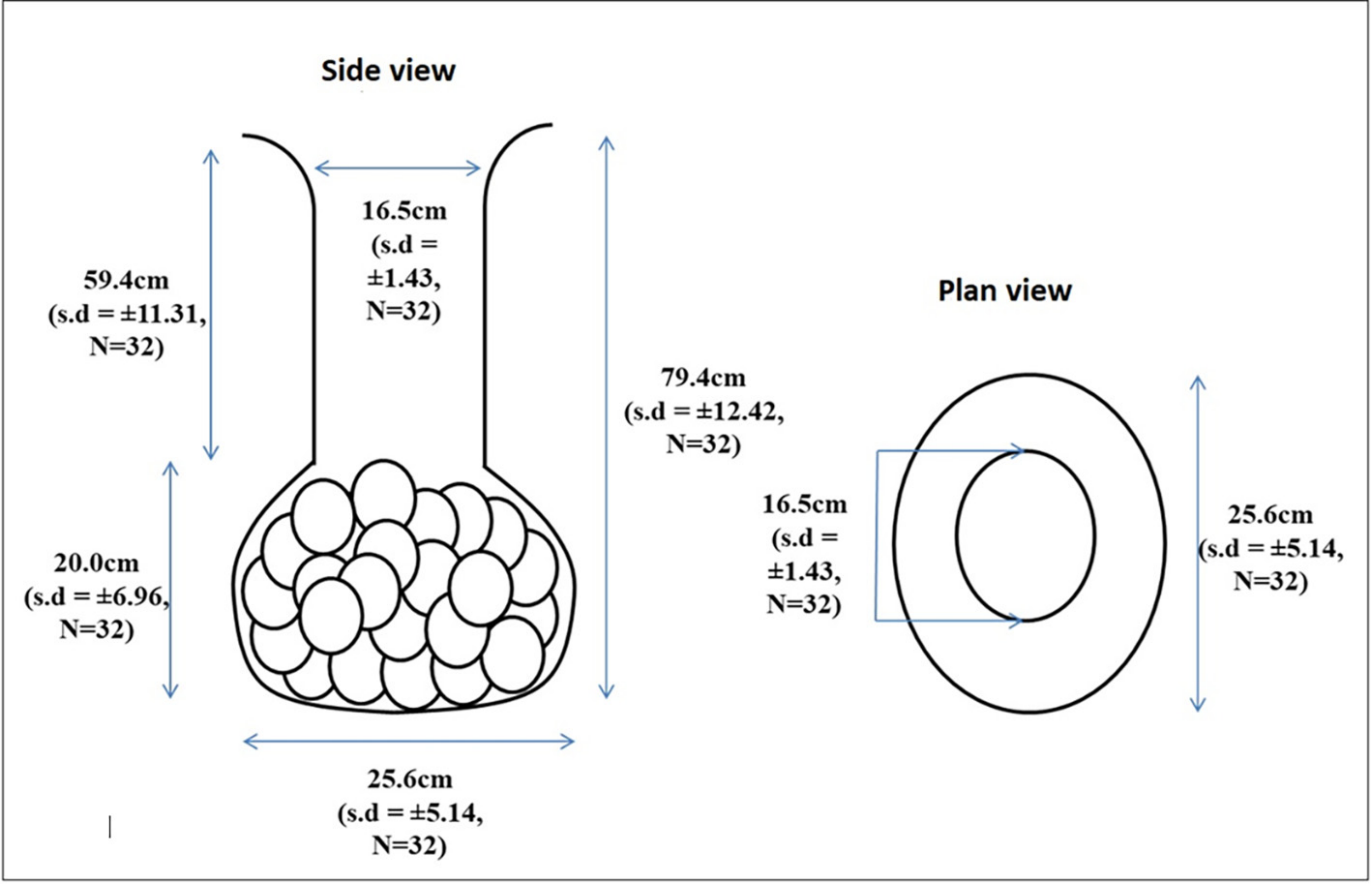
Mean nest shape and dimension for Chelonia mydas nesting in Chagar Hutang, Pulau Redang in 2017. The standard deviation (s.d) and sample sizes (N) are stated in parentheses.

The shape is illustrated using a 2D green turtle nest dimension (Fig. 8). From the figure, we can observe that the green turtle nest shape in Chagar Hutang has an oval bottom flask. The length of the bowl is not similar to the diameter of the bowl. Thus the measurements form an oval bowl shape.

**Fig. 9:**
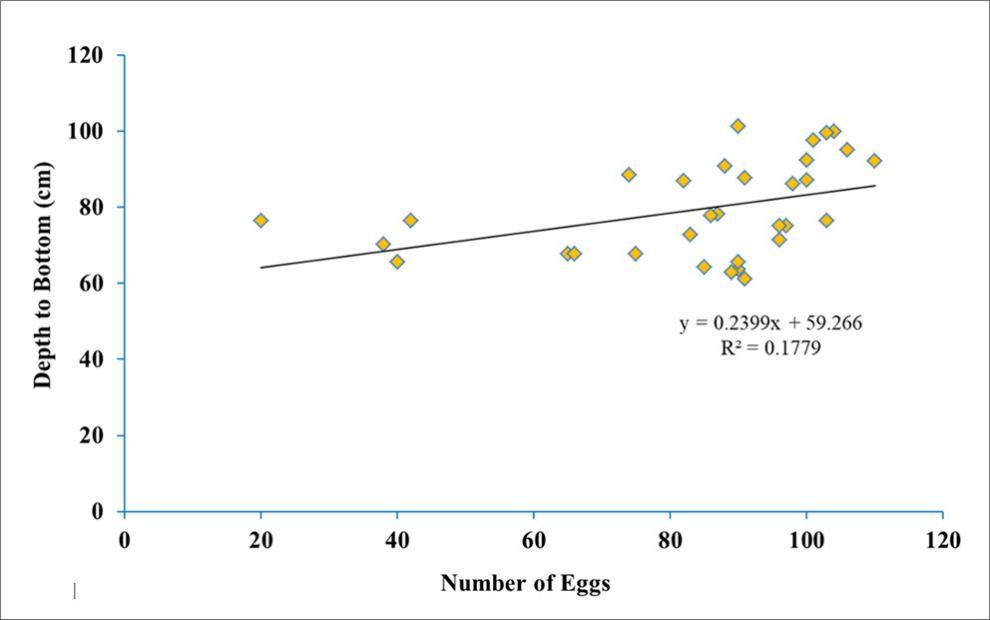
Linear regression graph between the number of eggs with the depth to the bottom of the green turtle nest in Chagar Hutang, Pulau Redang.

### 3.2 Relationship between nest depth and hatching success

The artificial nest (*ex-situ*) was constructed by SEATRU staff with a depth between 80 cm and 100 cm. The nest depth depended on the number of eggs and also the current ambient temperature. There is no significant difference in hatching success between *in-situ* and *ex-situ* methods in Chagar Hutang (t (31) = 1.12, p = 0.13).

Linear regression was conducted to observe the strength of the relationship between depth to bottom nest with the number of eggs. The results shows that the number of eggs have a significant results (f _(2, 29)_ = 0.04, p = 0.03, r^2^ = 0.202). Linear regression also revealed the relationship between depths to bottom nest with number of egg, f (1, 29) = 0.02, p = 0.04, r^2^ = 0.2 (Fig. 9). Therefore, the predictive equation of regression analysis is;

Y =mX+C

The m is the number of eggs coefficient (0.237), and the C is the intercept coefficient (59.327). Using this equation predictor, we could estimate the optimum nest depth for a certain number of green turtle eggs. This predictor could be implemented for ex-situ nests as well.

## 4 Discussion

CHTS implements both *in-situ* and *ex-situ* conservation method. The vulnerable nests were relocated to the middle of the beach or nearby the base camp. Nests located far from the basecamp are challenging to monitor by the staff and are most likely to be attacked by predators. At the nesting beach, several common predators recorded are; monitor lizard (Squamata: Varanidae), fire ants (Hymenoptera: Formicidae) and ghost crabs (Decapoda: Ocypodidae). The existence of barriers and predators are the factors that influenced the nest success (e.g., nesting success, hatching success, mortality rate and emergence success) of the green turtle (Nel et al., 2013, Zavaleta-Lizárraga and Morales-Mávil, 2013, Kelly et al., 2017). According to Ficetola (2007), beaches with barriers, for example, rocks, were reported to have high nesting abandonments. On both sides of Chagar Hutang beach (Sector 1/2 and 34/35) are rocky shores. From this study, both ends of the beach sites were recorded the lowest nesting frequency. These sectors were inappropriate for nesting and restricted the turtle from passing to certain parts of the beach.

Green turtle prefers a nesting site with fine sand (Zavaleta-Lizárraga and Morales-Mávil, 2013) and close to the vegetation (Kelly et al., 2017). According to Hays et al. (1995), sea turtle nesting is closely clumped near vegetation. Sector 16/17 and 30/31 are the most preferred area for nesting at CHTS Chagar Hutang. From observation, these sectors have fine sand and are have abundant vegetation. However, the preferences of the green turtle are inconclusive. According to Neeman et al. (2015), nesting near the vegetation has a high risk of invading roots and attacks from fire ants. Several studies suggested that sea turtle does not have any nesting site preferences, but instead scatter their nests randomly (Kamel and Mrosovsky, 2004). Therefore, an in-depth study regarding nesting and beach management should be conducted to have a firm agreement on this issue. Another point of a future study that could be explored is the sand particle size. We currently have anecdotal evidence from rangers and researchers that the sand particle at CHTS is much fine compared to the beaches on the mainland.

The reproductive output of green turtle at CHTS provides fundamental data to the conservation of this population in Terengganu. Studies regarding the nest morphology of sea turtle are scarce. There is no in-depth study regarding the nest shape of the green turtle to the best of our knowledge. However, several studies illustrate the nest dimension of other species; leatherback and flatback sea turtle. Koch et al. (2007) have illustrated the nest dimension of flatback turtles in Bare Sand Island, Australia. Koch et al. (2007) suggested that a flatback turtle has a cylindrical nest bowl with an average diameter of 28.9 cm, bowl depth of 15 cm and a total nest depth of 53.4 cm. However, the details regarding the study method were not well explained. Billes and Fretey (2001) also conducted a similar study with different sea turtle species, the leatherback. They used a casting method using thermocuring polyurethane foam to gain a 3D view of the leatherback nest dimension and determine the nest cavity's internal volume. The average nest depth of leatherback is 76 cm which the measurements allocate for the washbowl, laying well, and incubation chamber.

From this study, the average nest depth of green turtle at CHTS is 79.4 ± 12.42 cm, and the depth ranged from 61.1 to 101.2 cm. The average diameter of the nest bowl is 25.6 ± 5.14 cm with the height, 20 ± 6.96 cm, which gives the nest and oval-shaped bowl. Our results show that the green turtle nest have a round bottom flask shape. The average nest depth for green turtle is varied by region. The previous study stated that the nest depth range for green turtle is 40–100 cm (Booth and Freeman, 2006, Cheng et al., 2008, Gomuttapong et al., 2013). The nest depths are the critical factor for the success of the *ex-situ* conservation method (Martins et al., 2007). The researchers need to know the necessary information regarding the nest morphology of a species. Variation in nest depth influences the nest temperature, where the deeper nest generally cooler and more constant than the shallower nest (Broderick et al., 2000, Booth and Astill, 2001). Van de Merwe et al. (2005) stated that the sand temperature remains relatively constant over nesting season at places closer to the equator like Malaysia; the temperature difference between months was insignificant in Chagar Hutang. In this study, we initially recorded and tested the temperature effect; however, the results were insignificant. Hence, the nest depth is believed to influence the green turtle nest temperature. Nest temperature is more commonly related to the influence of the sex ratio of the hatchlings, incubation period, hatching success and also mortality rate of the green turtle.

The variation in nest depth is usually influenced by the female turtle size and the number of eggs laid. Several studies had proved that different sizes of females have different clutch size and numbers (Broderick et al., 2003). In this study, we did record the female turtle size. We found that there is a positive correlation between body size and the number of eggs. However, this portion of the study is currently included in another publication that is under review. For this manuscript, we choose to focus on the nest depth. Mortimer (1990) stated that the nest depth is a factor that influences the hatching success of sea turtle. According to Ekanayake et al. (2016), clutches with a nest depth between 60 and 100 cm had a higher hatching success than the shallower nest at Kosgoda. However, hatching success tends to show a negative correlation when the nest exceeded 100 cm. This is due to the less temperature variation and uniformity when the nest becomes deeper.

The nest depth could also influence the nesting beach's predation rate (Leighton et al., 2009). Nest predators are one of the significant factors in egg mortality for many buried nest organisms, particularly the reptiles (Spencer and Thompson, 2003). Zero maternal care after oviposition makes the eggs entirely dependent on the nest environment to survive. An increase of 15 to 45 cm in nest depth parallels the reduction of predation risk from 78.3% to 31.9% (Leighton et al., 2009). Many predators rely on their smell sense to detect the buried eggs. Therefore, deeper nests could obscure the olfactory cues of the predator (Stancyk, 1982, Cornelius, 1986, Leighton et al., 2009). At CHTS, the common predators found are ghost crabs, water monitor lizards and fire ants. Based on our results, we feel that it is essential for the ex-situ conservation method to apply the optimum depth by a green sea turtle to decrease the predator attack at the nesting area.

Besides predation, nest depth is also an essential factor influencing the hatching and emergence success of green turtle. There is a correlation between hatching success with the nest depth throughout this study. In general, deeper nest depth increases temperature stability and sand uniformity, favouring embryo development (Spotila et al., 1987, Miller, 1997, Tomillo et al., 2015). Successful emergence of the hatchlings is correlated with nest depth (Miller et al., 2003). According to Van De Merwe et al., 2005, Glen et al., 2005, deeper nests extend the time of hatchling emergence and have a higher risk in hatchlings mortality rate. Crawling out the nest demand much energy from the hatchlings (Brown et al., 2004). The hatchlings utilised half of the residual yolk, which fuels their energy metabolism for the emergence process (Rusli et al., 2016).

On the contrary, according to Martins et al., 2007, Marco et al., 2017, deeper nest have higher survival rates than the shallower nest, and deeper nests also enhanced the simultaneous emergence resulting in a higher number of hatchlings emerging at the same time. This behaviour can increase the hatchling survival rate by limiting the time for the predators to capture multiple preys. Therefore, the researcher must investigate the optimum depth of the nest correspond to the number of eggs to yield high hatching and emergence rate of the green turtle in Chagar Hutang.

### 4.1 Relationship between nest depth and the number of eggs of the green turtle in Chagar Hutang, Pulau Redang

The splitting clutch design method has been implemented in Malaysia, such as at Kerachut Turtle Conservation Centre (KTCC) (Sarahaizad et al., 2018) and several hatcheries in Malaysia (Ibrahim et al., 2002). This method was performed when Mortimer et al. (1994) stated that the hatching rate would increase by splitting and incubated the clutches into two equal half. However, this method has been challenged by Brown and Shine (2009), which does not agree with this conventional experimental. The splitting clutch method is believed to influence offspring size and capability. Rusli et al. (2016) stated that it is inappropriate for the clutch to be split into equal half; reducing the number of eggs will reduce the hatchlings energy reserves, crucial for their survival. Sarahaizad et al. (2018) supported this finding, which suggested the hatchery management to avoid the splitting clutch method and proposed the eggs to be incubated in a nest that duplicated the natural nest conditions. This is because naturally, the hatchling will combine their digging effort, which known as 'social facilitation', to crawl up and out the nest (Rusli et al., 2016), thus conserve their energy for further uses.

The nest depth that we used is quite different from the standard depth practised by the Department of Fisheries Malaysia, about 50–60 cm depth. However, we have to emphasise that we could not directly compare the effectiveness of such depth differences since we do not have access to the DOF data, and the experiment is conducted at different locale and time. This study has come out with a regression equation to improve the translocation method by predicting the optimum depth for a certain number of eggs of the green turtle in Chagar Hutang.

Y =0.2366X+59.3267

This regression equation derived from the in-situ data with a minimum parallax error. The *p* and F values for this equation indicate that this equation is stable to be implemented. Using the equation, 100 eggs have been tested to determine the optimum depth of the nest. The outcome shows the standard nest depth (80–100 cm) implemented by SEATRU staff correspond to the equation result where for 100 eggs, the depth must be 84 cm. The hatching rate between ex-situ and in-situ also does not show any significant difference. These findings show that the artificial nest at Chagar Hutang successfully resemble the natural nest. We would like to point out various debates on the differences between natural and artificial incubation for sea turtles (Stewart et al., 2020, Tanabe et al., 2021). It can be summarised that each nesting beach is unique on its own; there are confounding factors that might affect one specific place compared to another, such as the sand grain size (Stewart et al., 2020), the use of shades at the conservation centres (Reboul et al., 2021). There is one commonality in these studies; the conservation efforts are non-damaging to the survivability of these sea turtles.

Instead of using a fixed nest depth, we suggest that a calculation method is more effective and reliable for the translocation method and the SOP. Furthermore, this equation also can be implemented for the splitting clutch method to figure out the best nest depth for the eggs. However, the reliability and accuracy of the equation are limited. The equation perhaps can only fit for a specific range number of eggs. The accuracy of the estimate values does not guarantee any specific actual results. Therefore, continuous and extensive assessment of this issue should have actual optimum nest depth for the green turtle.

## 5 Conclusion

The nest translocation is a crucial method in conserving the population of the green turtle. Starting from placing the eggs into the basket, the journey from nesting beach to the relocated area, and the artificial nest's construction, these processes are crucial in determining the embryo development and hatching success of green turtle. The relocated nest must imitate the natural nest as close as possible, especially the nest depth. The survival of green turtle nesting is influenced by natural factors (e.g., temperature, rainfall, predators, vegetation and strong wave). Still, it also depends on the hatchery practices in understanding the nest morphology of green turtle and their implementation.

From this study, the green turtle prefers to nest in areas with high vegetation and fine sand particle. Furthermore, the green turtle also has an oval bottom flask shape, and the nest depth ranged from 61.1 to 101.1 cm. It is suggested that the equation method was used to gain a suitable depth instead of using the standard depth. However, the regression equation has a limit to 120–150 eggs, the maximum number of eggs generally laid by a green turtle.

## Declaration of Competing Interest

The authors declare that they have no known competing financial interests or personal relationships that could have appeared to influence the work reported in this paper.
